# What Endocrinologists Should Know About Iodine: Population Deficiency, Individual Excess and Misinformation in the United Kingdom

**DOI:** 10.1111/cen.70162

**Published:** 2026-05-17

**Authors:** Peter N. Taylor, Malcolm Prentice, Sarah Bath, Emilie Combet, Nadia Schoenmakers, Vicki E. Smith, Fraser W. Gibb, Nicola Zammitt, Onyebuchi Okosieme, Kristien Boelaert, John Lazarus, Mark Vanderpump

**Affiliations:** ^1^ Department of Immunology and Immunotherapy, School of Infection, Inflammation and Immunology, College of Medicine and Health University of Birmingham Birmingham UK; ^2^ Thyroid Research Group, Systems Immunity Research Institute Cardiff University School of Medicine Cardiff UK; ^3^ Nuffield Health Epsom UK; ^4^ Discipline of Nutrition, Exercise, Chronobiology and Sleep, Faculty of Health and Medical Sciences University of Surrey Guildford UK; ^5^ School of Medicine, College of Medical, Veterinary & Life Sciences University of Glasgow Glasgow UK; ^6^ School of Medical Sciences, Metabolism & Systems Science, College of Medicine and Health University of Birmingham Birmingham UK; ^7^ University/BHF Centre for Cardiovascular Science, Queen's Medical Research Institute, University of Edinburgh Edinburgh UK; ^8^ Edinburgh Centre for Endocrinology and Diabetes Royal Infirmary of Edinburgh Edinburgh UK; ^9^ Department of Applied Health Research, School of Health Sciences, College of Medicine and Health University of Birmingham Birmingham UK; ^10^ OneWelbeck Endocrine London UK

**Keywords:** Iodine deficiency, kelp, misinformation, pregnancy, public health nutrition, thyroid, United Kingdom

## Abstract

Iodine deficiency, once considered eliminated in the United Kingdom, has re‐emerged, particularly among women of reproductive age, in whom requirements are increased during pregnancy and lactation. The declining consumption of dairy and white fish, the emerging use of unfortified plant‐based milk alternatives, and the absence of a national iodisation policy have contributed to a return of mild to moderate iodine deficiency. Vegans and individuals avoiding dairy are at particular risk. Simultaneously, social‐media‐driven wellness trends promote ‘thyroid detoxes’ or ‘iodine resets’ and kelp‐based supplements that deliver supraphysiological iodine doses far exceeding recommended intake. Endocrinologists now face a dual challenge: widespread under‐recognition of population iodine insufficiency and a growing minority of patients presenting with iodine‐induced thyroid dysfunction. This review summarises current UK iodine status, the physiological and clinical relevance of iodine to thyroid and neurodevelopmental health, modern dietary risk factors and the clinical approach to those influenced by online misinformation. Routine dietary counselling and public health fortification represent cost‐effective interventions to prevent both extremes. Population‐based solutions would correct iodine status efficiently and align the UK with other high‐income countries.

## 1 Introduction

Iodine is an essential micronutrient required for thyroid hormone synthesis and normal neurodevelopment. Significant deficiency impairs thyroxine (T₄) and triiodothyronine (T₃) production, leading to hypothyroxinaemia, goitre, and—in pregnancy—irreversible neurocognitive harm to the developing foetus [1, 2]. Inadequate iodine intake impairs thyroid hormone production and can result in a spectrum of disorders collectively termed iodine deficiency disorders, ranging from goitre to irreversible brain damage [3, 4]. Globally, iodine deficiency remains the leading preventable cause of intellectual disability [1].

The World Health Organization (WHO) classifies population iodine sufficiency based on median urinary iodine concentration (UIC): 100–299 µg/L for non‐pregnant adults and 150–249 µg/L for pregnant women [5]. It should be noted that urinary iodine concentration is a population‐level indicator and is not intended for the assessment of iodine status in individual patients. The recommended dietary intake in the UK is 140 µg/day for adults, including during pregnancy and lactation [6, 7, 8]. The absence of recommended increments for pregnancy and lactation in the UK guidance is different to that of WHO or the European Food Safety Authority.

For practising UK endocrinologists, iodine has re‐entered significant clinical relevance—not only because of its importance to thyroid health and disease epidemiology [9] but also in view of our role in mitigating the risk of the widespread iodine deficiency in women of childbearing age [10] as well as addressing misinformation about excessive iodine supplements in autoimmune thyroiditis, and managing people with unusual iodine diets. This review provides a concise, evidence‐based overview of the current UK status, iodine physiology as well as the impact of supplements including potent and iodine detoxes.

### 1.1 Current UK Iodine Status and Dietary Advice

#### 1.1.1 From Accidental Sufficiency to Renewed Deficiency

For several decades, the UK was believed to be iodine‐replete, yet that status was achieved entirely by accident. During the 1940s and 1950s, the introduction of iodine‐based disinfectants (iodophors) in dairies and iodine‐supplemented cattle feed enriched the iodine content of milk, the combined effect being the reduction in endemic goitre without any government programme [11]. This ‘accidental fortification’ produced one of public health's quiet triumphs, but it also bred complacency. From the late 1980s onwards, organic herds were fed lower mineral supplements, and milk consumption declined [12]. Simultaneously, fish intake is low and plant‐based dietary patterns have expanded. The UK never pursued a universal salt iodisation or voluntary fortification policy [13, 14] and is therefore particularly vulnerable to changes in dietary iodine intake unlike the majority of other countries. Indeed, this situation is almost unique amongst high income countries.

In 2011, Vanderpump et al. [15] identified that iodine deficiency had reemerged as a public health issue in the UK. In a nationally representative sample of teenage schoolgirls, the study revealed a median UIC of 80 µg/L, well below the 100 µg/L threshold for adequacy in non‐pregnant populations, classifying the cohort as mildly iodine deficient. In the Avon Longitudinal Study of Parents and Children (ALSPAC), the median UIC during the first trimester of pregnancy was 91 µg/L (below the 150 µg/L threshold for pregnant populations), and children born to women with an iodine‐to‐creatinine ratio below 150 µg/g had lower verbal IQ and reading scores at 8‐9 years compared to those born with women with iodine‐to‐creatinine above 150 µg/g even after adjustment for multiple confounders [16].

Since 2013, the *National Diet and Nutrition Survey* (NDNS) has provided nationally representative urinary iodine data. The 2019–2023 survey reported a median UIC of 82 µg/L in women aged 16–49 years, with 30% of samples < 50 µg/L, well below the WHO threshold for adequacy [10]. Not only is this median UIC low, it has also shown a steady year‐on‐year decline, falling 29% among girls aged 11–18 years and 25% among adults aged 19–64 years since 2013 [10]. Recent data from Belfast report a concerning median UIC among pregnant women of 73 µg/L, despite self‐reported supplement use in more than half of participants [17]. Together, these findings demonstrate that mild to moderate iodine deficiency occurs across much of the female population, including those entering pregnancy. Of particular concern is the widening tail into moderately severe iodine deficiency, with an observed exponential relationship between severity and offspring IQ [1, 18].

Despite over a decade of consistent evidence for population iodine deficiency, the UK has not implemented salt iodisation. This reflects a combination of political inertia and institutional fragmentation: responsibility for fortification lies between the Department of Health, the Food Standards Agency and devolved administrations, with no single accountable lead. The Scientific Advisory Committee on Nutrition (SACN) ‘watching brief’ has recommended further research rather than action, and concerns about excessive sodium intake have made policymakers reluctant to endorse any salt‐based vehicle, despite evidence from other high‐income countries that iodine fortification can be achieved without increasing salt consumption [19].

Iodine status fluctuates daily, and urinary iodine concentration reflects only recent intake. Single spot or even 24‐h measurements are therefore unhelpful for individual diagnosis. Median values from large population samples are reliable, but for a single patient, dietary history provides more useful information. Individual testing should be reserved for research or for specific clinical protocols such as pre‐radioiodine therapy [19]. For population assessment, the World Health Organization recommends that median urinary iodine concentration estimates are derived from samples of at least 100 individuals. The UK population studies cited above, including the National Diet and Nutrition Survey, school‐based surveys, and pregnancy cohorts, meet or exceed this criterion.

### 1.2 Dietary Sources

In the UK, dairy products contribute 34% of total iodine intake in adults and 51% in children 4–10 years [10]. UK milk is a rich source of iodine, and has a concentration that is higher than that of milk from most other countries [20], meaning that the UK is heavily reliant on milk and dairy products as a source of iodine. White fish, such as haddock and cod, supply 150–300 µg per portion; and oily fish tends to have a lower iodine concentration [13]. Eggs provide about 20–25 µg each [21]. The most striking recent change in the UK diet is the rise of plant‐based milk alternatives. Sales have increased more than fivefold over the past decade, yet only 28% of milk alternatives are fortified with iodine, and just 6% of yoghurt alternatives are iodine‐fortified; without fortification, the iodine content of alternative products is low, around 2% of the cow's milk value [22, 23]. A 200 mL serving of cow's milk (i.e. a glass) provides around 60 µg of iodine, but an unfortified alternative would provide around 2 µg. Only a small proportion of retail salts are iodised, and ‘sea’ or ‘Himalayan’ salts contain negligible iodine [24].

Children and even adults with neurodiversity and highly restricted eating patterns represent an additional at‐risk group. In paediatric endocrine and general clinics, children who eat only a narrow range of ultra‐processed foods (e.g. crisps or chips‐only diets) may present with multiple micronutrient deficiencies, including iodine deficiency [25]. These cases underline the importance of clinicians enquiring about severely restricted diets when evaluating unexplained thyroid dysfunction or goitre in children and adolescents.

### 1.3 Multi‐Vitamins

Although iodine supplementation in pregnancy is cost saving even in mild to moderate deficiency [26] fewer than half of UK prenatal multivitamins include iodine, and awareness among healthcare professionals remains low [27]. Some women rely on kelp extract rather than the more ubiquitous potassium‐iodide form. Kelp (a type of seaweed) as foods or supplements are not recommended as an iodine source during pregnancy, because of the potential for iodine excess, but also the high variability and inconsistent transparency in their labelling (exact source of kelp, iodine content) [28]. If using kelp‐based products, switching to potassium iodide preparations is recommended. As the foetal thyroid begins to function only around week 14, supplementation should ideally start before conception [6]. Clinicians should therefore verify the exact product used and confirm that it provides approximately 150 µg of iodine daily in a stable iodide form.

SureStart and other NHS‐provided ‘free’ pregnancy vitamins [29] do not contain iodine, meaning that only women who purchase commercial products such as those listed in Box 1 receive appropriate supplementation. This creates a socioeconomic gradient in iodine intake during pregnancy—precisely the period when adequate maternal iodine is most critical for foetal neurodevelopment. Addressing this omission in publicly provided supplements represents a simple, equitable public‐health intervention.

Box 1 **Examples of UK prenatal or multivitamin products providing an appropriate iodine dose (product information accessed December 2025).**

- Pregnacare Original/Pregnacare Plus (Vitabiotics): 150 µg iodine as potassium iodide per daily dose.
- Seven Seas Pregnancy and Folic Acid Range (Seven Seas Ltd): 150 µg iodine as potassium iodide.
- **Oxford Vitality** Potassium Iodide 150 µg tablets—inexpensive.

Some other well‐known products do *not* contain iodine, while certain ‘natural’ or ‘vegan’ formulations use kelp powder with highly variable content. Clinicians should emphasise that reliable products specify both the iodine *dose* and *form* on the label, and that preparations using kelp or ‘seaweed extract’ are best avoided because of unpredictable iodine concentrations.

Endocrinologists should ask about milk type, fish intake and supplement use when assessing patients with subclinical hypothyroidism, women planning pregnancy, or adolescents following low‐dairy or vegan diets. Routine urinary‐iodine testing is not suitable for individual assessment of iodine status and can give misleading results; dietary history can instead identify those likely to be at risk of iodine deficiency. Incorporating brief iodine advice into thyroid and pre‐pregnancy consultations represents a cost‐free preventive step that may improve both endocrine and neurodevelopmental outcomes.

### 1.4 Practical Dietary Advice Endocrinologists Can Give

Adults require around 140 µg iodine per day, while pregnant and lactating women need approximately 200 µg [6]. Key counselling points for clinicians include:

- Maintaining regular dairy or iodine‐fortified plant drink alternative consumption.
- Consuming two portions of fish weekly, including at least one white fish.
- Including eggs can contribute a useful amount of dietary iodine.
- For vegans or people on dairy‐free diets, recommending a supplement providing 150 µg iodine (as potassium iodide or potassium iodate).
- Sea, rock, or Himalayan salts are not iodine sources.
- Begining supplementation before conception is recommended; foetal neurodevelopment depends on maternal thyroxine supply in the first trimester [6, 30].

### 1.5 Iodine and Thyroid Disease Epidemiology

The association between iodine intake and thyroid disease follows a classic *U*‐shaped curve [31]. Deficiency impairs thyroid hormone synthesis, causing compensatory TSH stimulation, thyroid hypertrophy, and, if prolonged, goitre and hypothyroidism. Severe deficiency produces frank hypothyroidism and obvious neurological impairment [4]. Iodine excess can precipitate hypo‐ or hyperthyroidism through failure to escape from the acute Wolff–Chaikoff block or through iodine‐induced hormone over‐production in autonomous tissue (Jod‐Basedow effect). Autoimmune thyroiditis displays a complex relationship with iodine. In very low‐intake regions, chronic iodine deficiency appears to suppress thyroid autoimmunity, while increasing iodine intake enhances immunogenicity of thyroglobulin and raises anti‐thyroid antibody prevalence [9, 31].

During Denmark's DanThyr iodisation programme, transient increases in anti‐thyroid peroxidase (TPO) positivity and autoimmune hypothyroidism were observed in middle‐aged women, followed by stabilisation once iodine status plateaued [32, 33]. This pattern suggests that iodine sufficiency unmasks pre‐existing susceptibility rather than generating new disease. The incidence of thyrotoxicosis rose by about 40% in the first 3 years after iodised‐salt introduction, concentrated in older adults with multinodular goitre [32]. The rise was temporary and outweighed by long‐term reductions in goitre prevalence, thyroid surgery and hypothyroidism rates. These data underscore that transient spikes in hyperthyroidism are a common and expected feature of successful correction using salt iodisation or equivalent fortification. Experience from Denmark and other European programmes shows that such effects are short‐lived and followed by substantial population benefit [32, 33, 34] as the burden of long‐term thyroid dysfunction from endemic goitre and nodular disease receeds.

Contemporary UK data place the population on the deficient limb of the U‐curve. Mild iodine insufficiency contributes to elevated thyroglobulin and subclinical hypothyroidism, particularly among women and adolescents. Endocrinologists should therefore consider minor TSH elevations in this context, differentiating dietary insufficiency from intrinsic autoimmune disease. The absence of a fortification policy leaves the population vulnerable to the long‐term consequences of deficiency—potential cognitive loss in offspring, goitre resurgence and potentially higher healthcare costs related to thyroid imaging and surgery. In iodine‐deficient settings, such as currently in the UK, serum thyroglobulin concentrations rise, reflecting thyroidal stimulation, while mean TSH drifts upwards without overt hypothyroidism [4].

Whilst severe iodine deficiency results in obvious neurological impairment, even mild deficiency during pregnancy is associated with subtle but measurable reductions in offspring IQ and reading performance [16, 35, 36]. Mild and moderate iodine deficiency appears to result in subtle, diverse and adverse neurological phenotypes rather than a classic phenotype. The risk operates primarily in the first trimester, when the foetal thyroid is not yet functional and relies entirely on maternal thyroxine. This is a particular issue as many pregnancies are unplanned, highlighting the importance of raising the population median iodine level to bring the tail above moderate insufficiency. Whilst randomised clinical trial data showing improvement in IQ with correcting mild iodine deficiency is lacking, there is evidence of supplementation effectiveness in moderate deficiency [37].

Economic analyses also consistently show iodine fortification to be one of the most cost‐effective public‐health interventions worldwide [4]. The Iodine Global Network estimates that universal salt iodisation costs approximately £0.02–0.05 per person per year, while the economic return typically exceeds £30 for every £1 invested [5, 38]. In Denmark, modelling associated with the *DanThyr* programme estimated net annual savings of at least €10–15 million from reductions in nodular thyroid disease, thyroid surgery and related investigations after the introduction of iodised salt [32, 33, 34, 39]. Additional gains from improved child cognitive development are expected but have not been fully quantified to date. Global analyses summarised by the World Health Organization indicate that each one‐point increase in population IQ is associated with substantial lifetime productivity gains, typically US$3000–6000 per child, equivalent to roughly £2300–4800 when adjusted to UK purchasing‐power levels. Although iodisation programmes may lead to a short‐term rise in thyrotoxicosis among older adults with multinodular goitre, experience from Denmark and other European countries shows that these transient costs are outweighed many times over by long‐term reductions in thyroid disease burden [32, 33]. The principal ongoing expense is population surveillance, which is modest, typically £150,000–300,000 per year in countries with monitoring systems comparable to the UK.

### 1.6 Diets, Supplements and Misinformation

The renewed interest in plant‐based and ‘clean‐eating’ lifestyles has created a perfect environment for iodine confusion. National marketing data show that roughly one‐third of UK households now purchase plant‐based milk, and sales have risen fivefold in a decade. In the Nottingham vegan cohort, the median UIC was only 30–40 µg/L—comparable to historically moderately deficient regions [40]. Similar results have been observed in analyses of vegan and vegetarian diets [41, 42]. The group at highest risk—young women—is the same group for whom iodine sufficiency is most critical for pregnancy and foetal brain development.

### 1.7 The Myth of ‘Natural’ Salts and Seaweed

The wellness movement has popularised ‘natural’ salts and seaweed as sources of trace minerals. In reality, virtually all UK retail salts—whether sea, rock, or Himalayan—contain negligible iodine (< 0.1 mg/kg), whereas iodised salt contains 20–40 mg/kg. Iodised salt is available to buy in the UK. Seaweed, by contrast, is a broad category of species which can contain enormous iodine concentrations, but with extreme variability between species. Nori provides ≈15–40 µg/g, while kombu and certain kelps exceed 2000 µg/g [43, 44, 45, 46]. Analyses of commercial macroalgae products in Europe found servings delivering 5–62,400 µg iodine [45]. Regular consumption of such products can easily exceed the European Food Safety Authority (EFSA) upper limit of 600 µg/day [47]. A summary of iodine supplements is shown in Table 1.

**Table 1:** Examples of iodine‐containing products and typical iodine content.

Product/Source	Typical iodine content per serving	Notes and clinical relevance
Kelp tea (infusion from kelp granules)	500–3000 µg per cup	Reported cases of thyrotoxicosis after daily consumption for several weeks. Iodine content depends heavily on kelp species and brew strength
Kelp tablets/‘sea‐moss’ capsules	1000–10,000 µg per tablet or capsule	Extreme variability; often unlabelled or inaccurate content. High risk of iodine‐induced hypo‐ or hyperthyroidism, particularly in autonomous or Graves' disease
Lugol's iodine (5%)	~6250 µg per drop	Historical antiseptic; sometimes promoted online as a ‘hyroid reset’. One drop ≈30–40 × the adult RNI
Seaweed snacks (nori sheets)	15–40 µg per sheet	Generally safe if eaten occasionally (e.g. sushi once weekly)
Kombu or kelp broth/soup	2000–10,000 µg per portion	Among the richest dietary sources; repeated use easily exceeds EFSA upper limit (600 µg/day)
Iodised table salt (where available)	20–40 µg/g	Predictable, safe source in fortified countries; rare in the UK
Prenatal multivitamin (with potassium iodide)	150 µg per daily dose	Provides safe, physiological supplementation; preferred form for deficiency prevention

Abbreviations: µg, micrograms; EFSA, European Food Safety Authority; RNI, reference nutrient intake.

Early reports of seaweed‐induced thyroid dysfunction appeared more than 4 decades ago and continue to accumulate [45]. Recent examples include women developing overt thyrotoxicosis after regularly consuming kelp tea [48] or kelp slimming diet [49]. Norwegian seaweed‐consumer studies found that habitual macroalgae eaters had high urinary iodine concentrations and a mixed pattern of thyroid‐function changes, while product analyses showed many single portions exceeding the 600 µg/day tolerable upper level and poor iodine labelling [45]. Some patients present with symptomatic thyrotoxicosis requiring β‐blockade or temporary antithyroid therapy, and hypothyroidism is also seen. Specifically for the UK, red seaweeds used in Welsh laverbread are generally lower in iodine than brown kelps, so occasional traditional consumption is unlikely to cause harm. The risk lies with frequent or concentrated ingestion, as well as with kelp‐based supplements, which should be avoided.

Mechanistically, excessive iodine transiently suppresses hormone synthesis via the acute Wolff–Chaikoff effect, whereas in autonomous or damaged thyroids it may instead trigger the Jod‐Basedow phenomenon. Consequently, sustained seaweed ingestion can produce either hypothyroidism or thyrotoxicosis, depending on individual gland physiology. Advising patients to avoid kelp tablets, ‘sea‐moss’ drinks, and high‐iodine detox teas is therefore a safe and clear message. Clinicians should advise complete avoidance of seaweed supplements and limit edible‐seaweed to stay within safe limits.

More concerningly online communities promote ‘iodine‐loading’ or ‘thyroid‐reset’ protocols advocating 12.5–50 mg iodine/day—100 to 300 times the Reference Nutrient Intake (RNI). Proponents cite early 20th‐century case series, ignoring later evidence of toxicity. Controlled studies show no metabolic benefit of high‐dose iodine in euthyroid individuals and a substantial risk of both hypothyroidism and autoimmune activation [2]. In Japan, where dietary iodine is high, habitual intakes above 1 mg/day correlate with elevated TSH and subclinical hypothyroidism [50]. The clinical challenge is not to confront patients but to re‐frame the concept: the thyroid requires iodine ‘not too little, not too much’, a message readily understood as the Goldilocks principle.

Short‐form video platforms have become a major route by which people, particularly those in their teens and 20 s, learn about thyroid disease. That is very close to the age group of concern in the UK—young women with falling iodine intake [51]. A 2024 cross‐sectional study of 200 TikTok and BiliBili videos on thyroid nodules found that the median DISCERN (quality of written health information) and GQS (quality of online health content) scores were only 3/5, and the authors concluded the overall quality and reliability were ‘unsatisfactory’; videos from thyroid specialists scored significantly better than those from influencers [52]. An analysis of Hashimoto's thyroiditis videos across YouTube, BiliBili and TikTok showed the same pattern: most videos were popular, but a high proportion lacked core clinical information, and very few mentioned when to seek medical care [53]. Common omissions were red‐flag symptoms, pregnancy considerations and the risks of self‐medicating with iodine.

Crucially, this is not just ‘low quality’—it changes behaviour. In a web‐based survey of 1172 US women aged 18–29, Kirkpatrick et al. [51] found that 58% had seen health‐related content on TikTok, and about one‐third reported changing what they took or bought (including supplements) as a result of videos. Many respondents stated that they could not determine whether creators were qualified or financially affiliated with the products.

The broader social‐media literature helps explain why this happens. Nickel et al. analysed almost 1000 Instagram and TikTok posts about popular medical tests and found that 84%–87% of posts were promotional, only ~6% mentioned harms, and influencers often had a commercial interest [54]. The same engagement mechanics apply to ‘thyroid reset’, ‘seaweed’ and ‘iodine deficiency’ videos — emotive storytelling and strong visual cues are promoted; balanced, guideline‐consistent information is not. Algorithms further amplify emotionally charged messages, meaning that objective rebuttals from credible sources rarely achieve similar reach. The result is a fragmented public narrative oscillating between deficiency panic and supplement excess. Clinicians increasingly encounter patients convinced either that iodine is a universal cure or that it is dangerous and best avoided.

For endocrinologists, the implication is that patients may arrive having seen: ‘Everyone is iodine‐deficient’, ‘Sea‐moss cures hypothyroidism’, ‘Doctors do not tell you about iodine because…’ Direct confrontation rarely changes belief. Communication research suggests that *affirm‐then‐add* strategies are most effective:

- Acknowledge truth: ‘You're right—iodine is essential for thyroid hormone production’.
- Add nuance: ‘But in the UK, most people need only 140 µg per day; more than 600 µg can actually switch the thyroid off’.
- Quantify and simplify: numbers and analogies are more persuasive than abstract caution.
- Provide credible resources: These include the BDA iodine factsheet (https://www.bda.uk.com/resource/iodine.html), British Thyroid Foundation (https://www.btf-thyroid.org/diets-and-supplements-for-thyroid-disorders), and peer‐reviewed educational material.

Building this brief counselling step into endocrine and pre‐pregnancy clinics can prevent both deficiency and iatrogenic excess. Some clinical encounters are shown in Box 2.

Box 2: **Practical clinical scenarios.**

**Table:** 

Scenario	Key considerations and guidance
28‐Year‐old vegan planning pregnancy	At high risk of deficiency. Advise daily multivitamin supplement containing 150 µg iodine as potassium iodide, and ensure use of fortified plant‐based milk. Begin before conception.
45‐Year‐old with subclinical hyperthyroidism taking kelp supplements	Likely iodine‐induced thyrotoxicosis. Stop supplements; reassess thyroid function after 4–6 weeks. If persistent, evaluate for autonomous nodule(s). Avoid further seaweed‐derived products.
Patient asking about an ‘iodine‐loading protocol’ from social media	Explain risks of high‐dose iodine (≥ 1 mg/day) and lack of benefit. Use ‘Goldilocks principle’ framing—too little or too much is harmful. Offer evidence‐based NHS and British Thyroid Foundation resources.

By contrast, deliberate iodine restriction (low iodine diet) is a validated short‐term measure before radioiodine (I¹³¹) therapy for differentiated thyroid cancer. Limiting iodine intake for 1–2 weeks enhances uptake by thyroid remnants and metastases [55, 56]. Patients avoid iodine‐rich foods—milk, fish, seaweed, iodised salt. Misinterpretation of this protocol online occasionally prompts patients with benign thyroid disease to self‐restrict chronically, risking deficiency. Clinicians should clarify that the low‐iodine diet is a short‐term and specialist‐supervised regimen.

## 2 Conclusions

Iodine deficiency has quietly re‐emerged in the United Kingdom after decades of neglect. Falling dairy and fish consumption, unfortified plant‐based products, and the absence of salt iodisation have undone the ‘accidental sufficiency’ once provided by the dairy industry. National surveys now show widespread mild deficiency among women and adolescents, with potential implications for thyroid function, pregnancy outcomes and child neurodevelopment. At the same time, public awareness remains poor, and misinformation on social media has distorted perceptions of both deficiency and toxicity.

From a clinical perspective, endocrinologists play a central role in identifying and addressing this reversible condition. Routine dietary questioning, targeted supplementation advice for women considering pregnancy, and cautious interpretation of thyroid‐function tests in the light of iodine status are simple but effective interventions. Awareness of emerging excess‐intake syndromes—from kelp supplements and sea‐moss drinks—is equally important. By framing iodine sufficiency as a component of thyroid optimisation rather than an esoteric nutritional issue, clinicians can normalise discussion within endocrine and obstetric practice.

At a population level, restoring iodine sufficiency will require joined‐up action between clinicians, scientists and policymakers. The UK's is one of only a few high‐income countries without a fortification policy. Universal salt iodisation or mandatory iodisation of table salt or fortification of plant‐based milks would likely close the gap swiftly and safely, as shown in other European nations [57]. Clear labelling of iodine content, routine surveillance through the NDNS, and coordinated education campaigns would sustain progress. The UK has the opportunity to convert a preventable micronutrient deficiency into a model public health success; the endocrine community is well‐placed to help through clinic‐based counselling, professional society advocacy for fortification, and public health campaigns.

## Data Availability

Data sharing not applicable to this article as no datasets were generated or analysed during the current study.
